# Your smile won’t affect me: Association between childhood maternal antipathy and adult neural reward function in a transdiagnostic sample

**DOI:** 10.1038/s41398-023-02364-y

**Published:** 2023-02-24

**Authors:** Katja I. Seitz, Kai Ueltzhöffer, Lena Rademacher, Frieder M. Paulus, Marius Schmitz, Sabine C. Herpertz, Katja Bertsch

**Affiliations:** 1 Department of General Psychiatry, Center for Psychosocial Medicine, Medical Faculty, Heidelberg University, Heidelberg, Germany; 2 Social Neuroscience Lab, Department of Psychiatry and Psychotherapy, University of Lübeck, Lübeck, Germany; 3 Center of Brain, Behavior and Metabolism (CBBM), University of Lübeck, Lübeck, Germany; 4 Department of Psychology, Ludwig-Maximilians-University Munich, Munich, Germany

**Keywords:** Human behaviour, Depression

## Abstract

Aberrant activation in the ventral striatum (VS) during reward anticipation may be a key mechanism linking adverse childhood experiences (ACE) to transdiagnostic psychopathology. This study aimed to elucidate whether retrospectively reported ACE, specifically maternal antipathy, relate to monetary and social reward anticipation in a transdiagnostic adult sample. A cross-sectional neuroimaging study was conducted in 118 participants with varying levels of ACE, including 25 participants with posttraumatic stress disorder (PTSD), 32 with major depressive disorder (MDD), 29 with somatic symptom disorder (SSD), and 32 healthy volunteers (HVs). Participants underwent functional magnetic resonance imaging during a monetary and social incentive delay task, and completed a self-report measure of ACE, including maternal antipathy. Neural correlates of monetary and social reward anticipation and their association with ACE, particularly maternal antipathy, were analyzed. Participants showed elevated activation in brain regions underlying reward processing, including the VS, only while anticipating social, but not monetary rewards. Participants reporting higher levels of maternal antipathy exhibited reduced activation in the brain reward network, including the VS, only during social, but not monetary reward anticipation. Group affiliation moderated the association between maternal antipathy and VS activation to social reward anticipation, with significant associations found in participants with PTSD and HVs, but not in those with MDD and SSD. Results were not associated with general psychopathology or psychotropic medication use. Childhood maternal antipathy may confer risk for aberrant social reward anticipation in adulthood, and may thus be considered in interventions targeting reward expectations from social interactions.

## Introduction

Adverse childhood experiences (ACE), such as abuse and neglect, are a common phenomenon, affecting approximately half of all adults in Europe and North America [1]. Associated with deleterious effects on mental health and psychosocial functioning [2–4], ACE increase the risk of various psychiatric disorders, including posttraumatic stress disorder (PTSD) [5], major depressive disorder (MDD) [6], and somatic symptom disorder (SSD) [7]. PTSD is characterized by intrusive re-experiencing of traumatic events, avoiding trauma-related stimuli, alterations of arousal and reactivity as well as changes in mood and cognition [8]. MDD is defined by depressed mood, loss of interest or pleasure, changes in appetite, sleep or psychomotor activity, fatigue, feelings of worthlessness, diminished ability to concentrate as well as recurrent suicidal ideation [8]. SSD involves one or more somatic symptoms which are distressing, persistent, and associated with excessive thoughts, feelings, or behaviors [8]. Gaining insight into mechanisms mediating the association between ACE and adult psychiatric disorders, such as PTSD, MDD, and SSD, is critical to develop effective early intervention and treatment strategies.

One potential mechanism linking exposure to ACE to adult psychopathology may be alterations in reward anticipation. Reward anticipation refers to the ability to anticipate or represent future incentives [9]. As an appetitive motivational process, reward anticipation plays a crucial role in adaptive decision-making and goal-directed behavior [10, 11]. On the neural level, reward anticipation elicits activity in a network of brain regions comprising the bilateral ventral striatum (VS), anterior cingulate cortex (ACC), anterior insula (AI), and supplementary motor area (SMA) [12–16]. Among these regions, the VS is of particular importance, encoding positive valence and the magnitude of upcoming rewards as a key mediator of reward prediction [12, 13].

Early experiences with primary caregivers, particularly mothers, shape reward anticipation throughout life [17]. Specifically, maternal warmth and encouragement in childhood and adolescence is perceived as integral for developing healthy reward processing [18, 19]. Maternal antipathy, a common type of ACE encompassing coldness and hostility [20, 21], may thus contribute to potentially debilitating aberrations in reward processing later in life. Initial empirical support for this notion comes from studies in children and adolescents suggesting maternal negative affect, lack of interpersonal affiliation, and lack of encouragement to be associated with reduced striatal reward responsiveness [18, 22, 23].

Interestingly, altered neural reward responses to reward anticipation relate to affective instability [24] and depressive symptom severity [25] in individuals with and without psychiatric disorders. In line with a dimensional perspective on psychopathology [26, 27], these findings suggest transdiagnostic deficits in reward system functioning, making it a promising treatment target beyond traditional nosological boundaries. Moreover, decreased activation in the reward circuitry, particularly the VS, during reward anticipation is consistently found in individuals with ACE (for reviews, see refs. [28–31]). In healthy adults, higher levels of ACE are associated with lower activation in brain regions processing reward during monetary reward anticipation, including diminished activity in the VS, ACC, AI, and middle frontal gyrus [32–34]. In adults with previous or current psychiatric disorders, findings have been inconclusive, with reduced [35] or increased [36] activation in brain reward regions during monetary reward anticipation associated with ACE. For example, reduced VS activation during reward anticipation is frequently found in individuals with MDD [25, 37–39]. Less is known about neural reward function in individuals with other disorders that have high prevalence rates of ACE, including PTSD [40, 41] and SSD.

Taken together, accumulating evidence indicates altered reward anticipation as sequelae of ACE in clinical and healthy samples. These findings may suggest exposure to ACE as a transdiagnostic risk factor for manifest and latent psychopathology in terms of altered neural reward responses to reward anticipation [42]. Nevertheless, previous studies on the association between ACE and neural correlates of reward anticipation in mental health conditions have been scarce and transdiagnostic studies spanning different psychiatric disorders have been missing so far. Moreover, despite previous studies proposing a strong influence of maternal parenting on neural reward processing [18, 22, 23], the specific association between maternal antipathy and reward anticipation has not been examined in adults with and without psychiatric disorders yet. Furthermore, previous research has focused on monetary rewards, although for humans social stimuli, such as smiling faces and positive feedback during social interactions, are one of the most powerful incentives motivating behavior [43, 44]. This is particularly relevant, as maternal antipathy more specifically shapes the formation of values and expectations of social reinforcers during development [17]. Thus, the question arises whether maternal antipathy may have a particularly strong association with social reward anticipation.

The aim of this study was to investigate the association between retrospectively reported ACE, specifically maternal antipathy, and neural correlates of monetary *and* social reward anticipation in a transdiagnostic sample. Following a dimensional approach, we recruited participants characterized by a broad range of ACE, both with psychiatric disorders known to show high prevalence rates of ACE—namely PTSD, MDD, and SSD—and healthy volunteers (HVs). Selecting PTSD, MDD, and SSD allowed us to capture common, highly comorbid disorders with distinct psychopathologies and strong associations with ACE [5–7]. Complying with the Research Domain Criteria’s (RDoC) recommendation [45], reward anticipation was examined using a modified version of the Monetary Incentive Delay Task [46], encompassing monetary and social rewards [47]. We hypothesized to find neural activation in the reward circuitry, particularly the VS, during monetary and social reward anticipation in the whole sample. We further hypothesized that higher levels of ACE and maternal antipathy in particular would be associated with diminished activation in brain regions underlying reward anticipation, particularly the VS, across all participants, while controlling for potentially confounding effects of clinical characteristics (i.e., general psychopathology, psychotropic medication). Since we expected a more pronounced association between maternal antipathy and social reward anticipation, monetary and social reward anticipation were analyzed separately. Finally, following the conceptualization of transdiagnostic relationships by Barch [48], in an additional exploratory analysis, we examined whether the hypothesized negative association between maternal antipathy and VS activation during social reward anticipation varies between groups of individuals with and without different psychiatric disorders.

## Materials and methods

### Participants

A total of 118 individuals with varying levels of ACE were included in this cross-sectional study. Originally, 136 individuals were recruited, with participants with psychiatric disorders (*n* = 100) being recruited through clinical referral from inpatient and outpatient units (*n* = 68) and advertisements (*n* = 32), and HVs (*n* = 36) being recruited through advertisements. Of the original sample, 5 had to be excluded due to neurological abnormalities, 1 due to technical problems, and 12 due to incomplete ACE data. The final sample comprised participants with a current and first lifetime DSM-5 diagnosis of PTSD (*n* = 25), MDD (*n* = 32), and SSD (*n* = 29), as well as HVs (*n* = 32). Despite the current lack of consensus regarding a priori sample size calculations in fMRI research, a recent publication [49] has deemed a sample size of 80 individuals or more to be sufficient for assessing brain-behavior correlations in task-related fMRI research.

Inclusion criterion for participants with psychiatric disorders was a DSM-5 diagnosis of either PTSD, MDD, or SSD, as assessed using the Structured Clinical Interview for DSM-5 (SCID-5 [50]). Given the high comorbidity rates among these three disorders, participants were allowed to fulfill diagnostic criteria for other psychiatric disorders including PTSD, MDD, and SSD as long as the “index diagnosis” could be established as the first lifetime and primary current diagnosis. The inclusion criterion for HVs was no current or past manifest psychiatric disorder according to the SCID-5 [50]. Exclusion criteria for all participants comprised: age younger than 18 and older than 60 years; any neurological disorders (e.g., epilepsy), a history of brain tumor, brain surgery or other major medical conditions; a current substance abuse assessed with a urine toxicology screening and the SCID-5 [50]; pregnancy; left-handedness; as well as standard magnetic resonance imaging (MRI) exclusion criteria (e.g., claustrophobia). Additional exclusion criteria for participants with psychiatric disorders included lifetime diagnoses of schizophrenia, schizoaffective or bipolar disorder, and self-reported severe substance use disorder in the prior 2 years as determined with the SCID-5 [50]. Current psychotropic medication was allowed for participants with psychiatric disorders only in terms of regular medication with antidepressants, antipsychotics (sleep-inducing effect only) and anticonvulsants (i.e., pregabalin, pain-relieving effect only).

The study was part of the German Research Foundation’s Research Training Group 2350 dedicated to investigating the impact of ACE on psychosocial and somatic conditions across the lifespan [51]. All participants provided written informed consent for the protocol approved by the Ethics Committee of the Medical Faculty of Heidelberg University and were reimbursed for their participation.

### Measures

#### Psychiatric disorders

Qualified diagnosticians (i.e., with at least a master’s degree in clinical psychology) assessed psychiatric disorders using the German version of the SCID-5 [50]. Diagnosticians received standardized diagnostic training before the beginning of the study. Inter-rater reliability was established by randomly selecting 12 video-taped diagnostic interviews which were rated by the head of the diagnostic unit and five independent raters, yielding an excellent [52] inter-rater reliability of *κ* = 1.00.

#### Adverse childhood experiences, including maternal antipathy

ACE were measured with the Childhood Experience of Care and Abuse Questionnaire (CECA.Q [20]; see Table 1). The CECA.Q, a retrospective self-report questionnaire, has been validated against the Childhood Experience of Care and Abuse (CECA) interview [53], an in-depth clinician-administered instrument which is considered a gold standard in retrospective assessment of ACE [54]. The CECA.Q covers lack of parental care, parental physical abuse, and sexual abuse with seven subscales.

**Table 1:** Sample characteristics.

Characteristic	Total (*N* = 118)		PTSD (*n* = 25)		MDD (*n* = 32)		SSD (*n* = 29)		HVs (*n* = 32)		Analysis	
	M	SD	M	SD	M	SD	M	SD	M	SD	*F* or χ^2^ or FET	*P*^a^
Age (years)	31.1	11.0	33.0	10.8	31.3	12.0	31.9	12.7	28.8	8.5	0.74	0.530
Educational level (years)	12.3	1.4	12.2	1.5	12.5	1.2	11.9	1.6	12.6	1.2	1.66	0.181
Psychological assessments												
CECA.Q antipathy mother	21.26	9.46	25.00	9.57	22.78	8.43	18.07	8.69	19.72	10.10	3.12	0.029
CECA.Q antipathy father^b^	22.90	9.17	24.32	10.24	24.81	8.70	20.50	8.73	21.87	8.90	1.38	0.251
CECA.Q neglect from mother	17.18	7.91	21.20	9.17	17.09	6.87	16.17	8.63	15.03	6.18	3.27	0.024
CECA.Q neglect from father^b^	23.19	8.74	26.68	9.82	22.06	7.07	23.15	9.85	21.52	7.95	1.92	0.130
CECA.Q physical abuse by mother	0.94	1.58	1.00	1.53	0.69	1.38	0.83	1.65	1.25	1.76	0.74	0.532
CECA.Q physical abuse by father	0.82	1.51	1.00	1.66	0.56	1.34	0.59	1.35	1.16	1.67	1.19	0.316
CECA.Q sexual abuse	1.10	1.99	2.96	2.72	0.63	1.54	0.59	1.21	0.59	1.41	11.79	<0.001
BSI Global Severity Index	0.92	0.66	1.25	0.69	1.40	0.52	0.74	0.41	0.33	0.36	29.81	<0.001
PCL-5 sum score	22.40	18.81	41.72	13.38	27.63	19.39	14.14	12.15	9.56	11.15	27.72	<0.001
BDI-II sum score	19.53	13.89	24.96	11.27	32.72	9.88	16.10	9.70	5.22	4.60	53.56	<0.001
SSD-12 sum score	18.66	11.64	19.36	8.46	17.66	10.63	30.52	5.91	8.38	8.33	34.16	<0.001
Psychotropic medication load	0.67	1.10	0.88	1.09	1.38	1.39	0.45	0.87	0	0	11.40	<0.001
fMRI task performance												
Hit rate (%)	66.02	6.79	63.89	8.04	66.93	6.39	65.76	6.59	67.01	6.18	1.26	0.290
Reaction time (ms)	285.24	39.73	302.06	49.51	288.01	38.66	277.55	38.75	276.29	28.89	2.55	0.060
Mean framewise displacement (mm)	0.12	0.08	0.12	0.10	0.12	0.09	0.12	0.07	0.11	0.07	0.06	0.979
Bilateral VS activation to social reward anticipation	0.17	0.97	0.54	1.65	0.09	0.55	0.10	0.62	0.02	0.81	1.58	0.199

Characteristic	Total (*N* = 118)		PTSD (*n* = 25)		MDD (*n* = 32)		SSD (*n* = 29)		HVs (*n* = 32)		Analysis	
	*N*	%	*N*	%	*N*	%	*N*	%	*N*	%	*F* or χ^2^ or FET	*P*^a^
Female	92	78.0	21	84.0	22	68.8	23	79.3	26	81.3	2.34^c^	0.504
Current medication												
Antidepressants	38	32.2	12	48.0	20	62.5	6	20.7	0	0	33.27^c^	<0.001
Antipsychotics^d^	5	4.2	2	8.0	3	9.4	0	0	0	0	4.89^e^	0.099
Anticonvulsants	2	1.7	1	4.0	0	0	1	3.4	0	0	2.53^e^	0.351
Current DSM diagnosis^f^												
PTSD	27	22.9	25	100.0	2	6.3	0	0	0	0		
MDD	45	38.1	7	28.0	32	100.0	6	20.7	0	0		
SSD	32	27.1	2	8.0	1	3.1	29	100.0	0	0		
Anxiety disorders	15	12.7	4	16.0	8	25.0	3	10.3	0	0		
Eating disorders	6	5.1	3	12.0	2	6.3	1	3.4	0	0		

*BDI-II* Beck Depression Inventory revised, *BSI* Brief Symptom Inventory, *CECA.Q* Childhood Experience of Care and Abuse Questionnaire, *FET* Fisher’s exact test, *HVs* healthy volunteers, *MDD* major depressive disorder, *PCL-5* PTSD Checklist for DSM-5, *PTSD* posttraumatic stress disorder, *SSD* somatic symptom disorder, *SSD-12* Somatic Symptom Disorder–B-Criteria Scale.

^a^Uncorrected for multiple testing.

^b^Data are missing for five participants (1 MDD, 3 SSD, 1 HV) who indicated that they did not grow up with their father/surrogate father.

^c^χ^2^ value.

^d^Sleep-inducing effect only.

^e^Fisher’s exact value.

^f^Participants were assigned to the group of individuals with PTSD, MDD, or SSD, if they were diagnosed with the respective disorder as first lifetime and current diagnosis at the time of study participation.

Maternal antipathy (i.e., antipathy from mother/surrogate mother) constitutes one of four subscales measuring lack of parental care, and covers maternal coldness, hostility, and rejection shown towards the child before the age of 18. Example items include „She made me feel unwanted” and “She was very critical of me” [20]. Maternal antipathy was assessed with eight items on a five-point Likert scale ranging from 1 (no, not at all) to 5 (yes, definitely). Subscale scores range from 8 to 40, with higher scores reflecting more severe maternal antipathy. A subscale-specific cutoff score of 25 has been recommended to indicate maternal antipathy in terms of ACE [20]. In the current sample, 41 out of 118 individuals (14 out of 25 PTSD, 13 out of 32 MDD, 5 out of 29 SSD, 9 out of 32 HVs) reported experiences of maternal antipathy above the subscale-specific cutoff score.

Beyond maternal antipathy, six additional CECA.Q subscales were used to determine other types of ACE, including further aspects of lack of parental care, physical abuse, and sexual abuse. Lack of parental care was assessed with three additional subscales, assessing paternal antipathy as well as neglect from mother/surrogate mother or father/surrogate father, respectively. Antipathy and neglect were measured with eight items each which were scored on a five-point Likert scale ranging from 1 (no, not at all) to 5 (yes, definitely). Corresponding to maternal antipathy, paternal antipathy comprises paternal coldness, hostility, and rejection shown towards the child before the age of 18. Neglect encompasses parental indifference regarding material, social, educational and emotional needs of the child. Subscale scores of lack of parental care range from 8 to 40, with higher scores reflecting more severe antipathy or neglect. Cutoff scores of 25 for paternal antipathy, 24 for neglect from father, and 22 for neglect from the mother have been proposed to indicate antipathy or neglect in terms of ACE [20]. Parental physical abuse was measured with two subscales assessing physical punishment by mother/surrogate mother or father/surrogate father, respectively, with four dichotomous items each (0 = no, 1 = yes). Subscale scores of parental physical abuse range from 0 to 4, with higher scores indicating more severe physical abuse. Sexual abuse in terms of unwanted sexual experiences with any person prior to the age of 18 was assessed with seven dichotomous items (0 = no, 1 = yes). Subscale scores of sexual abuse range from 0 to 7, with higher scores corresponding to more severe sexual abuse. A subscale-specific cutoff score of 1 has been suggested to indicate physical or sexual abuse in terms of ACE [20]. In the current sample, between 29 and 53 out of 118 individuals reported experiences of lack of parental care (i.e., paternal antipathy, neglect from mother or father), physical and sexual abuse above the subscale-specific cutoff scores.

#### Current symptom severity

Standardized self-report questionnaires were administered to assess current symptom severity (see Table 1). General psychopathology was measured with the Brief Symptom Inventory (BSI [55]), assessing 53 clinically relevant symptoms in the preceding seven days with 53 items on a five-point Likert scale ranging from 0 (not at all) to 4 (extremely). A BSI Global Severity Index (BSI GSI) of ≥0.62 is considered to indicate significant psychological distress [55]. PTSD symptom severity was measured with the PTSD Checklist for DSM-5 (PCL-5 [56]), assessing 20 PTSD symptoms in the last month with 20 items on a five-point Likert scale ranging from 0 (not at all) to 4 (extremely). Scores on the total scale range from 0 to 80, with higher scores reflecting more severe PTSD symptomatology. In the screening of PTSD, cutoff scores between 28 and 37 have been proposed [57–59]. Depressiveness was measured with the revised version of Beck’s Depression Inventory (BDI-II [60]), assessing 21 depressive symptoms in the preceding two weeks with 21 items on a four-point Likert scale. Total scores range from 0 to 63 with higher scores indicating more severe depressive symptoms. Criteria have been proposed to interpret the total score as reflecting mild (14–19), moderate (20–28), or severe (29–63) depression [60]. SSD symptomatology was measured with the Somatic Symptom Disorder–B-Criteria Scale [61], assessing the three psychological sub-criteria of SSD with four items each on a five-point Likert scale ranging from 0 (never) to 4 (very often). Total scores range from 0 to 48, with higher scores indicating a higher psychological symptom burden associated with somatic symptoms. Cutoff scores of 16 [62] or 23 [63] have been suggested to indicate a probable DSM-5 SSD diagnosis.

#### Psychotropic medication load

To control for possible confounding effects of the current medication status, a composite measure of psychotropic medication load was calculated for each participant, following procedures outlined in previous studies [24, 64, 65]. The daily dosages of each antidepressant, antipsychotic and/or anticonvulsant (i.e., pregabalin) taken regularly by an individual participant were each coded as absent = 0, low-dose = 1, or high-dose = 2, and summed up to calculate a composite measure of number and dosage of psychotropic medication (see Table 1). Antidepressants were categorized as low- (levels 1 and 2) or high-dose (levels 3 and 4) according to the criteria defined by Sackeim [66]. Antipsychotics were converted into chlorpromazine dose equivalents with low- or high-dose groupings corresponding to chlorpromazine dose equivalents equal or below, or above the mean effective daily dose of chlorpromazine [67, 68]. Pregabalin was coded as low- (≤300 mg) or high-dose (>300 mg) with reference to prior works [69, 70].

### Monetary and social reward fMRI task

Reward anticipation was examined using an adapted version of a well-established event-related incentive delay task [47], involving two types of rewards (i.e., monetary, social) and cues (i.e., reward cue, neutral cue; see Supplementary Fig. S1). The monetary and social incentive delay conditions were presented interleaved, in a pseudorandomized order, with 36 trials per condition, yielding a total of 72 trials. The primary outcome, neural activation to reward anticipation, was defined as the differential activation to anticipating rewarding (i.e., wallet with coins, happy face) as compared to neutral (i.e., wallet without coins, neutral face) outcomes, and was determined separately for both types of rewards.

In each trial, participants were required to press an MRI-compatible button as fast as possible with the index finger of their right hand as soon as a target symbol (i.e., yellow flash) appeared on the screen. To generate reward anticipation, the presentation of the target symbol was preceded by a cue signaling the reward that would be presented if the button was hit fast enough. Circle cues predicted two levels of monetary reward (i.e., an empty wallet or a wallet containing different amounts of coins), while square cues predicted two levels of social reward (i.e., a neutral or a happy facial expression). The reward level to be gained with a sufficiently fast reaction was indicated by the number of horizontal lines (i.e., one or three) in the middle of the circle or square cue, respectively. The stimuli for the social reward condition comprised photographs of seven individuals (three male, four female), displaying neutral or happy facial expressions, taken from the NimStim Set of Facial Expressions [71]. To generate outcome stimuli for the trials in which the participants reacted too slowly, photographs of wallets and facial expressions were graphically edited to blur out any object or facial features while keeping size and luminance stable.

Each trial started with the presentation of one of four cues (1000 ms), followed by a blank screen (2250–2750 ms), the target symbol (100 ms), and a fixation cross (400 ms). Feedback informing the participants about their reaction time (“outcome”) consisted of the presentation of a clear or blurry picture of the reward indicated by the preceding cue (2500 ms). Each trial ended with an inter-trial interval (2000–5000 ms), during which another fixation cross was displayed.

Prior to entering the scanner, participants performed at least six practice trials to familiarize themselves with the experiment. Participants were informed that their task performance in the scanner had no influence on their financial compensation. In the scanner, participants performed another six practice trials before the main experiment to calculate their individual mean reaction times. If participants reacted too slowly (>500 ms) during the six practice trials in the scanner, two additional practice trials were presented. Task difficulty was standardized to a hit rate of approximately 66% for all participants by adjusting the reaction time window, in which participants had to press the button, to their individual mean reaction times on a trial-by-trial basis. If participants hit the button fast enough (i.e., within their individual reaction time window), they saw clear pictures of wallets or facial expressions, depending on the cue preceding the target symbol (hit trials). If participants hit the button too slowly or before they saw the target symbol, they saw blurry pictures of wallets or faces (miss trials).

After the main experiment, participants were asked to rate each outcome stimuli (i.e., wallets and faces) with regard to how rewarding it was to them on a scale from 1 (not rewarding at all) to 9 (very rewarding) outside of the scanner. Stimulus presentation and recording of reaction times and reward ratings inside and outside of the scanner were performed using the software Presentation (Neurobehavioral Systems, Albany, CA, USA).

### fMRI acquisition parameters

Magnetic resonance imaging was performed on a 3.0 Tesla Siemens Tim Trio fMRI scanner equipped with a 32-channel head coil. Functional images were acquired using a BOLD-sensitive T2-weighted echo-planar imaging (EPI) sequence (repetition time [TR] = 2340 ms, echo time [TE] = 26 ms, flip angle = 80°; matrix: 96 × 96, field of view [FOV]: 220 × 220 mm, in-plane resolution: 2.3 mm, slice thickness: 2.3 mm, no interslice gap, 40 axial slices). In addition, isotropic high-resolution (1 × 1 × 1 mm) T1-weighted coronal-oriented structural images were recorded using a magnetization-prepared rapid gradient echo (MPRAGE) sequence. Experienced neuroradiologists reviewed all scans to exclude clinically relevant abnormalities which led to the exclusion of *n* = 5 participants.

### Data analysis

*Behavioral data* (i.e., hit rates, reaction times, post-fMRI reward ratings) were subjected to 2 (reward type: monetary, social) × 2 (reward level: reward, neutral) repeated-measures analyses of variance (ANOVAs) using IBM SPSS 26. The significance level was set at *P* < 0.05.

*Imaging data* were preprocessed and analyzed with Statistical Parametric Mapping (SPM12; https://www.fil.ion.ucl.ac.uk/spm/) running on MATLAB R2019a (The MathWorks, Natick, MA). To account for T1 saturation effects, the first five volumes of each participant were discarded. Image preprocessing followed standard routines of SPM12 and included slice-time correction, realignment, spatial normalization to the Montreal Neurological Institute (MNI) template, and smoothing with a Gaussian Kernel of 8.0-mm full-width at half-maximum (FWHM). Due to discomfort, one participant discontinued with the fMRI session before MPRAGE anatomical images could be acquired. For this participant, spatial normalization was realized with the mean EPI image instead of the MPRAGE image. Following Power et al. [72, 73], the framewise displacement between subsequent functional volumes was computed, based on the six rigid motion parameters estimated during the realignment step (see Table 1).

Preprocessed images were subjected to first-level general linear model (GLM) estimation. For each participant, a GLM was defined, including nine delta function regressors for the task conditions (four cues, four outcomes in hit trials, one for all outcomes in miss trials), which were convolved with the standard SPM hemodynamic response function, and six head motion parameters, extracted from the realignment procedure. GLM contrast images were derived at the first level by assessing neural activation to reward vs. neutral cues (i.e., monetary: wallet with vs. without coins; social: happy vs. neutral face, respectively).

First, at the second level, differential contrast images were entered into a 2 (reward type: monetary, social) × 2 (cue type: reward, neutral) full-factorial model to investigate neural activation during reward anticipation in the whole sample (i.e., task activation analyses). One individual regressor was added per participant as a second-level covariate of no interest to explicitly model the individual mean neural response to reward and neutral cues in the monetary and social condition [74].

Second, multiple linear regression analyses were performed to examine the association between neural activation during monetary and social reward anticipation, respectively, and maternal antipathy in the whole sample. Age, sex, years of education, general psychopathology (BSI GSI), and psychotropic medication load were added as second-level covariates of no interest to control for possible confounding effects of demographic and clinical characteristics. Corresponding multiple linear regression analyses were conducted to investigate the association between neural activation during monetary and social reward anticipation, respectively, and the additional six CECA.Q subscales.

Region-of-interest (ROI) and whole-brain analyses were conducted for both task activation and multiple linear regression analyses. Considering the central role of the VS in reward anticipation, ROI analyses were focused exclusively on this brain region. Following previous studies [12, 64, 75], 8-mm-spheres were centered on MNI coordinates in the left (*x* = −10, *y* = 10, *z* = −2) and right (*x* = 12, *y* = 14, *z* = −4) VS, and used as a bilateral a priori mask. Exploring further relevant areas, whole-brain analyses were performed. Significance was set at cluster-level *P* < 0.05, familywise error (FWE) corrected, with a single-voxel threshold *P* < 0.001 for whole-brain results, and additionally small-volume corrected for ROI results. Moreover, in the more exploratory multiple linear regression analyses with the additional six CECA.Q subscales, both ROI and whole-brain results were corrected for multiple testing using a Bonferroni-adjusted significance level of 0.05/6 = 0.008.

Finally, a moderation analysis was performed to explore whether the association between retrospectively reported maternal antipathy (independent variable) and VS activation to social reward anticipation (dependent variable) was moderated by diagnostic group (moderator). This exploratory moderation analysis was conducted using the PROCESS macro (version 3.5) [76] implemented in SPSS. Contrast estimates of the bilateral VS mask [12, 75] for social reward anticipation (social reward cue >neutral cue) were extracted with MarsBar (http://marsbar.sourceforge.net/) (see Table 1). Bootstrapping (20,000 resamples) was employed to estimate the 95% confidence intervals (95% CI) of the moderating effects of diagnostic group.

## Results

### Behavioral results

Participants achieved hit rates of ~66% (*M* = 66.0%, *SD* = 6.8%) due to standardization procedures (see Table 1). Reaction times did not differ significantly between reward type (*F*[1,117] = 0.73, *P* = 0.393) or level (*F*[1,117] = 0.26, *P* = 0.612). After the fMRI task, participants rated social stimuli (neutral and happy faces) as more rewarding than monetary stimuli (wallets with and without coins; *F* [1,117] = 104.82, *P* < 0.001) and rewarding stimuli (happy faces, wallets with coins) as more rewarding than neutral stimuli (neutral faces, wallets without coins; *F* [1,117] = 335.72, *P* < 0.001) (see the Supplementary File).

### Neural activation during reward anticipation

In the ROI analyses, activation within the bilateral VS mask was found only during social, but not during monetary reward anticipation (see Supplementary Table S1; social reward anticipation, right VS, *k* = 2; left VS, *k* = 3). Similarly, the whole-brain analyses revealed elevated activation in the brain reward circuitry for social, but not monetary reward anticipation (see Supplementary Table S1). Anticipating social rewards elicited widespread activation, encompassing the bilateral dorsal ACC, AI, SMA, putamen, right pallidum, thalamus, precuneus, middle frontal gyrus, bilateral occipital lobe, fusiform gyrus, and calcarine sulcus. Anticipating monetary rewards yielded activation only in the bilateral occipital lobe, fusiform gyrus, and right calcarine sulcus.

### Association of neural activation to reward anticipation with maternal antipathy

**Fig. 1:**
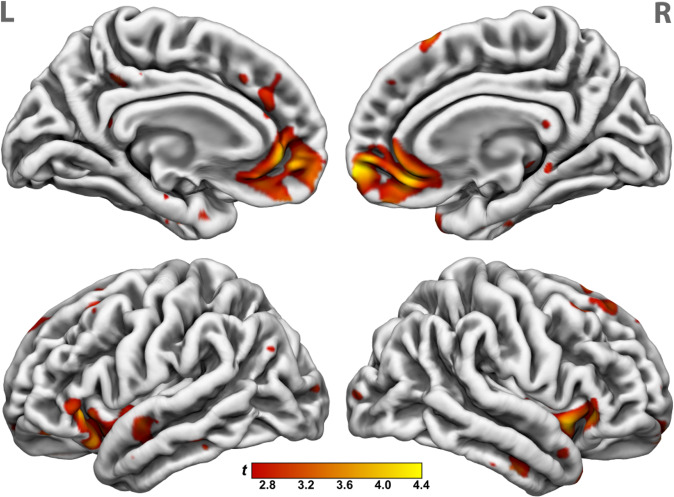
Association between childhood maternal antipathy and neural activation to social reward anticipation. *Note*. Whole-brain analyses indicated childhood maternal antipathy was associated with decreased activation during social reward anticipation in the ventromedial prefrontal cortex, bilateral anterior insula, and superior frontal sulcus. The four clusters survived corrections for multiple comparisons at *P* < 0.001, uncorrected and *k* > 320 corresponding to *P* < 0.05, FWE correction. The t-map of the regression analysis controlling for age, sex, years of education, general psychopathology, and psychotropic medication load was thresholded at *P* < 0.005 for displaying purposes and the color gradient depicts the respective *t*-values of the maternal antipathy regression weights.

During social reward anticipation, participants with higher levels of maternal antipathy showed reduced activation in key regions of the reward network, including the VS (ROI analysis) and the bilateral ACC, AI, ventromedial prefrontal and orbitofrontal cortex, and superior temporal gyrus (whole-brain analysis; see Table 2 and Fig. 1). No association was found between maternal antipathy and neural activation to monetary reward anticipation (ROI and whole-brain analyses). No association was found between the other six CECA.Q subscales and neural activation to neither monetary nor social reward anticipation (ROI and whole-brain analyses).

**Table 2:** Negative association of neural activation to social reward anticipation and childhood maternal antipathy.

Region	Hemisphere	*P*_*FWE*_^a^	Cluster size, k	*T*	*Z*	MNI peak voxel		
						*x*	*y*	*z*
Whole-brain results								
Anterior cingulate cortex, medial frontal gyrus, ventromedial prefrontal and orbitofrontal cortex	Left/right	<0.001	930	5.01	4.74	4	58	−4
				4.86	4.62	−2	46	−4
				4.59	4.38	6	34	−12
Middle and superior frontal gyrus	Left	0.004	320	4.95	4.70	−12	28	46
				4.45	4.26	−28	16	44
				3.84	3.71	−6	24	40
Insula, inferior frontal gyrus, superior temporal gyrus	Left	<0.001	524	4.67	4.45	−36	28	−6
				4.23	4.07	−40	−6	−12
				4.17	4.01	−38	20	−20
Insula, inferior frontal gyrus, superior temporal gyrus	Right	<0.001	509	4.31	4.14	42	14	−10
				4.18	4.02	46	24	−6
				4.13	3.97	38	28	−4
Region-of-interest results								
Ventral striatum	Left	0.024	14	3.69	3.57	−12	16	−6

*FWE* familywise error.

^a^Threshold set at *P*_*FWE*_ < 0.05.

### Moderation of association between maternal antipathy and VS activation to social reward anticipation

**Fig. 2:**
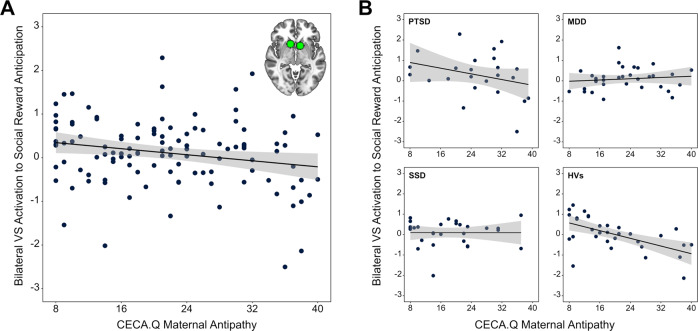
Moderation of association between childhood maternal antipathy and bilateral ventral striatal activation to social reward anticipation. **A** Association between childhood maternal antipathy and bilateral ventral striatal (VS) blood oxygen level-dependent (BOLD) response to social reward vs. neutral anticipation in the whole transdiagnostic sample after exclusion of one outlier. **B** Group affiliation moderates the association between childhood maternal antipathy and bilateral VS BOLD response to social reward anticipation with significant negative associations found in participants with posttraumatic stress disorder (PTSD) and healthy volunteers (HVs), and no association found in participants with major depressive disorder (MDD) and somatic symptom disorder (SSD). Please note that the scatterplots depict associations after the exclusion of one outlier. Please refer to Supplementary Fig. S2 for the corresponding scatterplots including the outlier.

After the extraction of the contrast estimates for bilateral VS activation to social reward anticipation, one participant with PTSD was identified as being an outlier (i.e., contrast estimates exceeding 3 *SD* of the sample’s mean). Diagnostic group significantly moderated the association between maternal antipathy and bilateral VS activation during social reward anticipation, both before and after the post-hoc exclusion of the outlier (with outlier: adjusted *R*^2^ change = 0.09, *F*[3,110] = 4.40, *P* = 0.006; without outlier: adjusted *R*^2^ change = 0.08, *F*[3,109] = 3.37, *P* = 0.021). Simple slopes revealed significant negative associations between maternal antipathy and bilateral VS activation to social reward anticipation in PTSD (with outlier: *b* = −0.71, 95% CI −1.06 to −0.35; *P* < 0.001; without outlier: *b* = −0.33, 95% CI −0.64 to −0.02; *P* = 0.035) and HVs (with outlier: *b* = −0.45, 95% CI −0.74 to −0.15; *P* = 0.003; without outlier: *b* = −0.45, 95% CI −0.69 to −0.21; *P* < 0.001) while no association was found for MDD (with outlier: *b* = 0.07, 95% CI −0.28 to 0.43; *P* = 0.684; without outlier: *b* = 0.07, 95% CI −0.22 to 0.36; *P* = 0.619) and SSD (with outlier: *b* < −0.01, 95% CI −0.36 to 0.36; *P* = 0.993; without outlier: *b* < −0.01, 95% CI −0.30 to 0.29; *P* = 0.992) (see Fig. 2 for the results without the outlier and Supplementary Fig. S2 for the results with the outlier). Furthermore, including general psychopathology (BSI GSI) and psychotropic medication load as covariates in the initial exploratory moderation analysis to control for possible confounding effects of clinical characteristics did not change the significance of the results (see the Supplementary File).

## Discussion

This study revealed a negative association between retrospective self-reports of childhood maternal antipathy and VS activation during social reward anticipation in a transdiagnostic sample. According to an exploratory moderation analysis, this was only the case in HVs and participants with PTSD, but not in those with MDD or SSD. Therefore, the current study provides initial evidence for differential mechanisms in the relationship between maternal antipathy, a specific, but common type of ACE, and altered neural reward responses to social reward anticipation.

In the current sample, higher levels of retrospectively reported childhood maternal antipathy were associated with decreased activation in key components of the brain reward network during social reward anticipation. By showing that a particular type of ACE, maternal antipathy, was related to diminished activity in the left VS during social, but not monetary, reward anticipation, our a priori ROI analysis adds to previously reported effects of maternal parenting on striatal reward responsiveness in children and adolescents [18, 22, 23], and indicates that maternal antipathy may attenuate social reward expectations. Furthermore, we found maternal antipathy to be associated with reduced responsivity in the bilateral ventral ACC, (anterior) insula, ventromedial prefrontal and orbitofrontal cortex, and superior temporal gyrus during social reward anticipation. As central nodes of the salience network, the ACC and AI are considered crucial for detecting motivationally meaningful stimuli and facilitating goal-directed behavior [77–79]. Specifically, the ventral (i.e., subgenual and perigenual) ACC and AI have been implicated in social decision-making, signaling social saliency [80], and prediction errors during social interactions [81]. While activation of the ventromedial prefrontal and (medial) orbitofrontal cortex has mainly been linked to reward consumption [12, 14, 15], medial prefrontal activity has also been positively associated with novelty seeking during anticipation of emotionally salient stimuli [82]. Finally, the superior temporal gyrus has been implicated in audiovisual emotional processing which is of particular importance for our everyday social interactions [83]. Consistent with our results, adverse maternal behavior has been associated with reduced attention to salient social cues [84], less novelty seeking [85], and functional aberrations in neural reward processing [18, 22, 23, 86]. Together, these findings suggest detrimental effects of maternal antipathy on reward system functioning with potentially negative consequences for daily subjective well-being [87]. Considering our and previous results, one intriguing explanation could be that early experiences of cold or hostile mothers might dampen the rewarding aspects of social interactions early in life with long-term implications for social reward anticipation in adulthood [23]. Due to the cross-sectional nature of this study, however, our findings can only be understood as a first indicator of retrospectively reported childhood maternal antipathy contributing to altered activation in the brain reward circuitry during social reward anticipation and require replication in longitudinal studies.

In line with previous research [13, 88], social reward anticipation elicited robust activation in several core reward regions, including the VS. Contrary to previous meta-analytic findings [13], however, monetary reward anticipation did not yield comparable activation in this circuitry. Consistent with these neural findings, participants rated social incentives as more rewarding than monetary incentives in the post-fMRI ratings. One reason for this preference of social incentives could be that participants received financial compensation for their participation independent of their task performance. In accordance with recent research [89], anticipating primary, immediate social rewards such as approvingly smiling faces may have thus elicited stronger activation in the brain reward circuitry than anticipating secondary, symbolic monetary rewards which did not lead to actual financial gain after the fMRI task.

Can we characterize the relationship found between maternal antipathy and VS activation during social reward anticipation as transdiagnostic according to Barch [48]? Pursuing this question, we detected significant associations only in HVs and participants with PTSD, but not in those with MDD and SSD. Of note, participants with PTSD reported significantly higher levels of maternal antipathy than participants with SSD, and participants with PTSD and HVs were characterized by a descriptively higher dispersion of both maternal antipathy scores and contrast estimates of bilateral VS activation to social reward anticipation than those with MDD and SSD. Extending prior research in HVs [33, 34] and PTSD [90], our findings suggest that these individuals may be more sensitive to maternal antipathy inducing striatal hyporesponsiveness to anticipating social rewards than individuals with MDD and SSD. While in participants with PTSD, this negative association may partly relate to the more pronounced severity of early maternal antipathy and possibly a high proportion of participants with complex PTSD, in HVs who do not present with a manifest psychiatric disorder it may reflect latent vulnerability to future mental health problems [42]. Nonetheless, more transdiagnostic, particularly longitudinal studies on the association between specific types of ACE and neural responses to reward anticipation are needed to allow for strong conclusions about diagnosis-specific associations.

Several limitations should be acknowledged: First, despite previous research indicating robust activation of the brain reward circuitry during monetary reward anticipation [13, 15, 16], this pattern was not replicated in our study. Speculatively, this may be owing to our sample’s higher appreciation of social incentives and lack of performance-based financial compensation. Since we did not want our participants to equate social rewards in the scanner with monetary rewards outside of the scanner, we refrained from performance-based financial compensation which might have led to these inconsistent results. Second, retrospective recall of specific types of ACE via self-report questionnaire is prone to memory biases and reflects subjective experiences of rather than actual exposure to ACE [91, 92]. Since our cross-sectional design does not allow for causal inferences, future longitudinal studies should investigate whether prospective measures of maternal antipathy are also associated with altered neural reward responses to reward anticipation in adulthood. Third, considering previously reported sex-dependent effects on the association between maternal affiliation and reward processing dysfunction [22], the disproportionate number of female participants in our sample limits generalizability to individuals of male or non-binary gender.

In conclusion, this study indicates differential associations between self-reported childhood maternal antipathy and social reward anticipation in individuals with and without major psychiatric disorders. By shifting the focus from composite measures of ACE to maternal antipathy, our study provides more precision as to which type of ACE should be considered in transdiagnostic neuroimaging studies of (social) reward anticipation. Future studies should also assess mechanisms promoting resilience (e.g., social support [93]) to further advance our understanding of the complex relationship between different types of ACE, psychiatric disorders, and altered reward anticipation. Ultimately, gaining a better understanding of these differential mechanisms may inform tailoring therapeutic interventions targeting reduced interest in pleasurable social activities to vulnerable individuals.

## Supplementary information

Supplement
