# Supplementary material for: Your smile won’t affect me: Association between childhood maternal antipathy and adult neural reward function in a transdiagnostic sample
Source: Transl Psychiatry. 2023 Feb 24;13:70. doi: 10.1038/s41398-023-02364-y (PMC9958053; doi:10.1038/s41398-023-02364-y)
Supplement: Supplementary file 1 — Supplement [file 41398_2023_2364_MOESM1_ESM.docx]

**Supplemental Information**

**Results**

**Behavioral Results**

Due to standardization procedures, hit rates did not differ significantly between reward type (*F*[1,117]=0.10, *p*=.755) or level (*F*[1,117]=0.00, *p*=.948), and reward type and level did not interact significantly (*F*[1,117]=1.53, *p*=.218). Participants were instructed to press the button as soon as the target appeared on the screen, regardless of the preceding cue. Consequently, concerning reaction times, besides no significant main effects of reward type or level described in the main manuscript, no significant interaction between reward type and level emerged (*F*[1,117]=2.77, *p*=.099). With regard to post-fMRI reward ratings, besides significant main effects of reward type and level described in the main manuscript, a significant reward type by level interaction emerged (*F*[1, 117]=34.04, *p*<.001), with happy as compared to neutral facial expressions gaining higher reward ratings than wallets with as compared to without coins.

**Moderation of Association Between Maternal Antipathy and Ventral Striatal Activation to Social Reward Anticipation Controlled for General Psychopathology and Psychotropic Medication Load**

After including general psychopathology (Brief Symptom Inventory Global Severity Index, BSI GSI) and psychotropic medication load as covariates in the initial moderation model, diagnostic group still significantly moderated the association between maternal antipathy and bilateral VS activation during social reward anticipation (adjusted *R^2^* change=.09, *F*[3,108]=4.24, *p*=.007). Simple slopes still revealed significant negative associations between maternal antipathy and bilateral VS activation to social reward anticipation in PTSD (*b*=-0.64, 95% CI -0.99 to -0.28; *p*<.001) and HVs (*b*=-0.40, 95% CI -0.70 to -0.11; *p*=.008) while no associations were found for MDD (*b*=0.12, 95% CI -0.25 to 0.48; *p*=.527) and SSD (*b*=0.04, 95% CI -0.32 to 0.40; *p*=.828).

**Figure S1.** *Monetary and Social Incentive Delay Task*


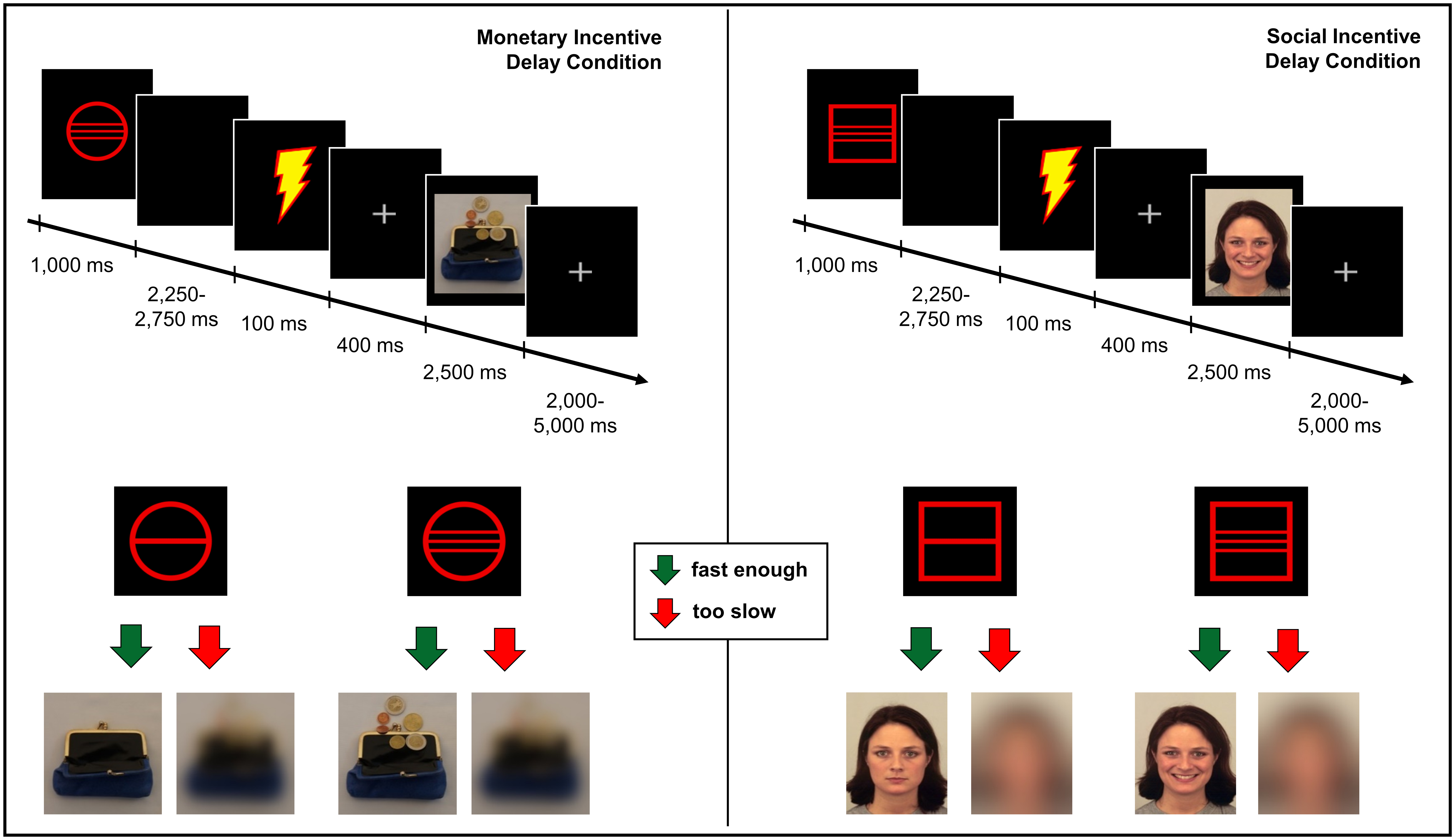


*Note.* Visualization of the Monetary and Social Incentive Delay Task, modified in accordance with Spreckelmeyer et al.^1^

**Figure S2.** *Moderation of association between childhood maternal antipathy and bilateral ventral striatal activation to social reward anticipation including one outlier*


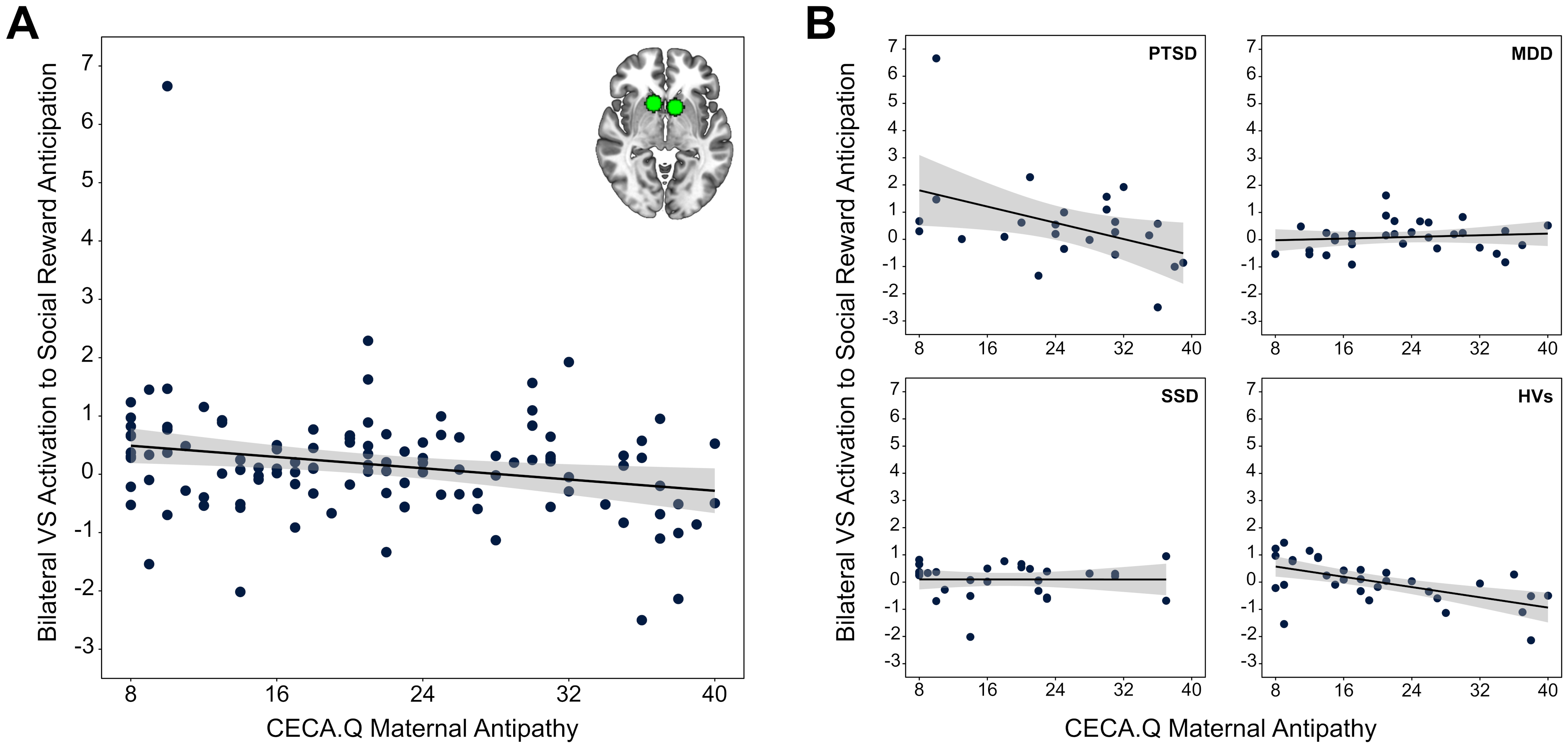


*Note.* A. Association between childhood maternal antipathy and bilateral ventral striatal (VS) blood oxygen level-dependent (BOLD) response to social reward vs. neutral anticipation in the whole transdiagnostic sample. B. Group affiliation moderates the association between childhood maternal antipathy and bilateral VS BOLD response to social reward anticipation with significant negative associations found in participants with posttraumatic stress disorder (PTSD) and healthy volunteers (HVs), and no association found in participants with major depressive disorder (MDD) and somatic symptom disorder (SSD).

**Table S1.** *Neural activation during reward anticipation*

|  |  |  |  |  |  | MNI peak voxel | | |
| --- | --- | --- | --- | --- | --- | --- | --- | --- |
| Region | Hemisphere | *p_FWE_^a^* | Cluster size, k | *T* | *Z* | x | y | z |
| *Monetary reward anticipation (monetary reward cue > neutral cue)* | | | | | | | | |
| Whole-brain results | | | | | | | | |
| Middle and inferior occipital gyrus, fusiform gyrus, calcarine sulcus | Right | < .001 | 884 | 7.07 | 6.83 | 20 | -98 | 2 |
|  |  |  |  | 4.65 | 4.57 | 28 | -72 | -8 |
|  |  |  |  | 4.02 | 3.97 | 28 | -40 | -18 |
| Middle and inferior occipital gyrus | Left | < .001 | 552 | 6.69 | 6.48 | -18 | -98 | -4 |
|  |  |  |  | 3.23 | 3.20 | -30 | -74 | -12 |
| Fusiform gyrus | Left | .004 | 345 | 5.32 | 5.21 | -30 | -56 | -12 |
|  |  |  |  | 4.99 | 4.90 | -32 | -48 | -18 |
| Region-of-interest results | | | | | | | | |
| Ventral striatum | no significant results | | | | | | | |
|  | | | | | | | | |
| *Social reward anticipation (social reward cue > neutral cue)* | | | | | | | | |
| Whole-brain results | | | | | | | | |
| Middle and inferior occipital gyrus, fusiform gyrus, calcarine sulcus | Left/Right | < .001 | 7057 | 8.63 | Inf. | 22 | -98 | 2 |
|  |  |  |  | 8.40 | Inf. | -20 | -98 | -4 |
|  |  |  |  | 7.05 | 6.81 | -24 | -88 | -8 |
| Supplementary motor area, dorsal anterior cingulate cortex, medial and superior frontal gyrus, left precentral gyrus | Left/Right | < .001 | 3106 | 5.18 | 5.08 | -6 | -4 | 66 |
|  |  |  |  | 4.81 | 4.73 | -6 | 4 | 52 |
|  |  |  |  | 4.80 | 4.72 | -44 | -6 | 44 |
| Putamen, pallidum, anterior insula, thalamus | Right | < .001 | 597 | 4.91 | 4.83 | 16 | -6 | 2 |
|  |  |  |  | 4.23 | 4.18 | 8 | -14 | 6 |
|  |  |  |  | 4.10 | 4.05 | 26 | -2 | 10 |
| Middle frontal gyrus, superior frontal junction | Right | .006 | 313 | 4.83 | 4.75 | 36 | -10 | 48 |
|  |  |  |  | 4.01 | 3.96 | 46 | -2 | 56 |
|  |  |  |  | 3.80 | 3.76 | 46 | -12 | 58 |
| Superior temporal gyrus | Left | .026 | 227 | 4.55 | 4.48 | -48 | -38 | 12 |
|  |  |  |  | 4.44 | 4.37 | -40 | -34 | 18 |
|  |  |  |  | 3.71 | 3.67 | -32 | -26 | 12 |
| Precuneus | Right | .018 | 248 | 4.40 | 4.34 | 14 | -32 | 56 |
|  |  |  |  | 3.66 | 3.62 | 20 | -34 | 62 |
|  |  |  |  | 3.56 | 3.52 | 6 | -36 | 54 |
| Putamen, anterior insula | Left | .014 | 265 | 4.37 | 4.31 | -30 | 14 | 12 |
|  |  |  |  | 3.78 | 3.74 | -24 | 10 | 0 |
|  |  |  |  | 3.33 | 3.30 | -38 | 10 | 8 |
| Region-of-interest results | | | | | | | | |
| Ventral striatum | Right | .047 | 2 | 3.26 | 3.23 | 18 | 12 | -8 |
|  | Left | .044 | 3 | 3.23 | 3.20 | -10 | 6 | -2 |

*Note*. FEW = familywise error; Inf. = infinite.

^a^ Threshold set at *p_FWE_*<.05.

**References**

1 Spreckelmeyer, K. N., Krach, S., Kohls, G., Rademacher, L., Irmak, A., Konrad, K. *et al*. Anticipation of monetary and social reward differently activates mesolimbic brain structures in men and women. *Soc Cogn Affect Neurosci* 2009; **4**: 158-165.
